# Age-Dependent Decline in Salinity Tolerance in a Euryhaline Fish

**DOI:** 10.3389/fragi.2021.675395

**Published:** 2021-06-09

**Authors:** Mayu Inokuchi, Yoko Yamaguchi, Benjamin P. Moorman, Andre P. Seale

**Affiliations:** ^1^ Department of Aquatic Bioscience, Graduate School of Agricultural and Life Sciences, University of Tokyo, Tokyo, Japan; ^2^ Institute of Agricultural and Life Sciences, Academic Assembly, Shimane University, Matsue, Japan; ^3^ Hawai‘i Institute of Marine Biology, University of Hawai‘i, Kaneohe, HI, United States; ^4^ Department of Human Nutrition, Food and Animal Sciences, University of Hawai‘i at Mānoa, Honolulu, HI, United States

**Keywords:** osmoregulation, osmoreception, fish, age, survival, salinity tolerance, gill, prolactin

## Abstract

Euryhaline teleost fish are characterized by their ability to tolerate a wide range of environmental salinities by modifying the function of osmoregulatory cells and tissues. In this study, we experimentally addressed the age-related decline in the sensitivity of osmoregulatory transcripts associated with a transfer from fresh water (FW) to seawater (SW) in the euryhaline teleost, Mozambique tilapia, *Oreochromis mossambicus*. The survival rates of tilapia transferred from FW to SW were inversely related with age, indicating that older fish require a longer acclimation period during a salinity challenge. The relative expression of Na^+^/K^+^/2Cl^−^ cotransporter 1a (*nkcc1a*), which plays an important role in hyposmoregulation, was significantly upregulated in younger fish after SW transfer, indicating a clear effect of age in the sensitivity of branchial ionocytes. Prolactin (Prl), a hyperosmoregulatory hormone in *O. mossambicus*, is released in direct response to a fall in extracellular osmolality. Prl cells of 4-month-old tilapia were sensitive to hyposmotic stimuli, while those of >24-month-old fish did not respond. Moreover, the responsiveness of branchial ionocytes to Prl was more robust in younger fish. Taken together, multiple aspects of osmotic homeostasis, from osmoreception to hormonal and environmental control of osmoregulation, declined in older fish. This decline appears to undermine the ability of older fish to survive transfer to hyperosmotic environments.

## Introduction

The maintenance of body fluid osmolality and cellular sensitivity to osmotic and endocrine stimuli are fundamental components of homeostasis that become compromised with age in vertebrates. Osmoreception, the first step in osmoregulation, involves the integration between cellular mediators of osmotic changes, and effectors, typically hormones, capable of acting systemically to restore salt and water balance (Grau et al., 1994; Bourque and Oliet, 1997; Seale et al., 2012b). Osmoregulation, in turn, depends on a balance between the intake and output of ions and water. In humans, aging is often characterized by reduced homeostatic capacity, and disturbances in sodium and water balance (Rolls and Phillips, 1990; Pomatto and Davies, 2017). A number of studies have shown age-related differences in thirst, hypothalamic cellular sensitivity, and osmoregulatory endocrine responses, especially those related to vasopressin release (Robertson and Rowe, 1980; Baylis, 1987; Mack et al., 1994; Lee et al., 1998; Thunhorst et al., 2010; Thunhorst et al., 2011). Dehydration with aging has been attributed to a loss in the ability to concentrate urine (O'Neill and McLean, 1992; Dmitrieva and Burg, 2011) and to a decrease in thirst (Kenney and Chiu, 2001). Furthermore, cellular senescence in osmoregulatory tissues, such as the kidney, is accelerated due to high interstitial NaCl concentration and contributes to the aging-related impairment of salt and water balance (Dmitrieva and Burg, 2007). While some studies have employed fish models to examine how senescence may relate to loss in osmoregulatory capability (Jeffries et al., 2011), little is known on the effects of age on the physiological mechanisms underlying the capacity of organisms to face salinity challenges. While terrestrial vertebrates are constantly exposed to the threat of dehydration due to high respiratory water loss, fishes are more vulnerable to osmotic changes because their body fluids are only separated from water of varying salinities by the thin respiratory epithelia of the gills (Takei et al., 2014). In teleost fish, the gill is considered to be a primary site for monovalent ion regulation, a function that is performed by the kidney in mammals (Evans et al., 2005). To the extent that both organs share many of the molecular mediators of ion transport, studies addressing age-dependent osmoregulatory responses of the gill may provide insight into similar mechanisms in humans. Model organisms with compact life spans that live in direct contact with aquatic environments and possess the physiological capacity to tolerate changes in salinity offer unique access for investigating the effects of aging on the endocrine and environmental control of osmoregulation.

Euryhaline teleost fish such as Mozambique tilapia, *Oreochromis mossambicus*, possess the capacity to tolerate a wide range of salinities. Mozambique tilapia have evolved in continually changing environments that are characterized by tidal changes in salinity spanning fresh water (FW) and seawater (SW) and are able to modify the function of the cells and tissues that regulate salt and water balance (Inokuchi et al., 2015; Moorman et al., 2014; Seale et al., 2019). Prolactin (Prl) is responsible for a variety of physiological actions in vertebrates including fish, amphibians, reptiles, and mammals (Bern and Nicoll, 1968). In fish, the osmoregulatory function of Prl is well established. Without the pituitary gland, euryhaline fish will not survive in FW unless it has received Prl replacement therapy (Pickford and Phillips, 1959; Dharmamba et al., 1967), a seminal finding that led to the continued characterization of the Prl cell as a model osmoreceptor (Grau and Helms, 1990; Seale et al., 2006; Seale et al., 2020). Prl secretion from the tilapia pituitary responds directly to the extracellular osmolality which it regulates (Grau et al., 1981; Seale et al., 2002). Once released into circulation, Prl acts on a number of osmoregulatory epithelia, such as the kidney, intestine, and gills, to promote ion uptake (Breves et al., 2011; Seale et al., 2014). In peripheral tissues, Prl’s actions are largely mediated through the regulation of genes involved in the transport of water and ions, including the Na^+^/Cl^−^ cotransporter (Ncc), Na^+^/K^+^/2Cl^−^ cotransporter (Nkcc), and Na^+^/K^+^-ATPase (Nka) (Breves et al., 2010; Tipsmark et al., 2011; Inokuchi et al., 2015). In gill, the genes encoding these effectors of osmoregulation are also directly regulated by changes in extracellular osmolality (Inokuchi et al., 2015). Inasmuch as osmoregulation is affected by age, we hypothesized that the ability of fish to survive a direct transfer from FW to SW environment decreases in older animals due to the attenuation of their responsiveness to osmotic and endocrine stimuli. Specifically, we addressed whether there is a difference in the osmotic sensitivity of Prl cells and the osmoregulatory response of gills to Prl between young and old fish.

## Materials and Methods

### Experimental Animals

Mozambique tilapia yolk sac fry were collected from broodstock tanks maintained in FW at the Hawaii Institute of Marine Biology (Kaneohe, HI). The fry were kept in 75-l glass aquaria supplied with circulating FW until yolk sac absorption was complete. The fry were then combined into one 75-L aquarium containing FW. Water temperature was maintained at 25 ± 1°C in all tanks. The fish were exposed to a 12L:12D cycle. Fish were fed crushed Silver Cup trout chow (Nelson and Sons, Murray, UT) *ad libitum* daily, except during both experimental trials, when they were fasted. Prior to survival trials, fish were transferred to outdoor tanks (700 l) with a continuous flow of FW at 25 ± 1°C under a natural photoperiod. Tilapia, aged 1, 4, 6, and more than 24 (>24) months (mo), were used for transfer experiments. All experiments were conducted according to the principles and procedures approved by the Institutional Animal Care and Use Committee, University of Hawaii.

### Direct Transfer Experiment

Twenty Mozambique tilapia of each age were kept in four separate tanks containing FW. The approximate average weight of 1-, 4-, 6-, and >24-mo-old fish were 1.0 ± 0.1, 7.5 ± 0.4, 26.4 ± 1.3, and 278.3 ± 22.1 g, respectively. At the onset of the experiment, the FW source was shut off and SW (34 ppt) was added to the tanks, such that fish were fully transitioned to full-strength SW by 1 h. Tanks were checked every 30 min over the following 24 h to monitor survival.

### Rate of Transfer Experiment

Forty Mozambique tilapia of each age were kept separately in four tanks containing FW. Three FW tanks were switched to SW by adding full-strength SW at different flow rates, taking 1, 3, and 12 h for complete salinity transfers. Survival was monitored for 24 h following SW transfer by checking the tanks every 2 h.

### Effects of Salinity on Branchial Gene Expression

FW-acclimated fish (mixed sex), aged 4 mo and >24 mo, were transitioned to SW over a 12-h period. Control fish were kept in FW for the duration of the experiment. From each of the four experimental tanks, the gill arches of 10 fish were sampled at the end of the 24 h period following completion of transfer.

### Dispersed Prolactin Cell Incubations

To examine the effects of age on tilapia Prl cell osmosensitivity, we compared hyposmotically-induced Prl release from dispersed Prl cells of FW-acclimated tilapia, aged 4 mo (8.9 ± 0.6 g, *n* = 10), and >24 mo (307.9 ± 22.6 g, *n* = 24), as previously described (Seale et al., 2012a; Yamaguchi et al., 2018). Briefly, after removal of pituitaries from adult tilapia, *rostral pars distalis* (RPD) were dissected and pooled in PBS (330 mOsm/kg), diced into pieces, and treated with 0.125% trypsin (Sigma, St. Louis, MO) in PBS. After terminating the trypsin treatment by the addition of trypsin inhibitor (0.125% w/v in PBS; Sigma), the cells were harvested and cell viability was determined using the trypan blue (Sigma) exclusion test. Cell yield was estimated using a hemocytometer. The cells were then resuspended in 355 mOsm/kg incubation medium, plated on 96-well plates at the density of 16,000 cells/well, and incubated at 26°C under saturated humidity. The cells were preincubated for 1 h in 355 mOsm/kg medium prior to incubation in hyperosmotic (355 mOsm/kg) and hyposmotic (300 mOsm/kg) media. The media samples were collected by 1 h of incubation and stored at −20°C, prior to analysis by radioimmunoassay.

### Effects of Prolactin on Cultured Gill Filaments

To identify direct effects of Prl on branchial gene transcript levels, we cultured filaments from the second and third gill arches of male and female FW-acclimated tilapia, aged 4 mo (10 and 12 g) and >24 mo (598 and 377 g), following Watanabe et al. (2016). Excised gill arches were first washed in sterilized balanced salt solution (BSS: NaCl 120 mmol/l; KCl 4.0 mmol/l; MgSO_4_ 0.8 mmol/l; MgCl_2_ 1.0 mmol/l; NaHCO_3_ 2.0 mmol/l; KH_2_PO_4_ 0.4 mmol/l; Na_2_HPO_4_ 1.3 mmol/l; CaCl_2_ 2.1 mmol/l; and Hepes 10 mmol/l; pH 7.4) and then incubated in 0.025% KMnO_4_ for 1 min. After a second wash in BSS, gill filaments of >24-mo-old fish were cut in half to minimize the effect of size. In a preliminary experiment, we confirmed that there were no differences in the expression of target genes between whole gill filaments and those cut by half (data not shown). Individual gill filaments were then cut sagittally under a dissecting microscope, and placed in 24-well plates (Becton, Dickinson and Company, Franklin Lake, NJ) containing Leibovitz’s L-15 culture medium (Gibco–Thermo Fisher Scientific, Waltham, MA). The culture medium was supplemented with 5.99 mg/l penicillin and 100 mg/l streptomycin (Sigma), adjusted to 330 mOsm/kg, and sterilized with a 0.2-μm filter. Two gill filaments were placed in each well, which contained 500 μl culture medium supplemented with 0, 1, and 5 μg/ml of ovine Prl (Sigma) (*n* = 4 for 4-mo-old fish and n = 8 for >24-mo-old fish). After overnight incubation (18 h) at 26°C, gill filaments were frozen in liquid nitrogen and stored at −80°C prior to RNA extraction and gene expression analyses.

### RNA Extraction and Real-Time Quantitative PCR

For each sample, total RNA was extracted using Tri-Reagent (Molecular Research Center, Cincinnati, OH), and then reverse transcribed using a cDNA Reverse Transcription Kit (Applied Biosystems–Thermo Fisher Scientific), according to the manufacturers’ protocols. The quantitative real-time PCRs (qRT-PCRs) were set up as previously described (Moorman et al., 2014; Inokuchi et al., 2015). Briefly, 200 nmol/l of each primer, 1 μl cDNA, and 7.5 μl of SYBR Green PCR Master Mix (Applied Biosystems) were added to a 15-μl final reaction volume. The following cycling conditions were employed for all assays: 2 min at 50°C, 10 min at 95°C followed by 40 cycles at 95°C for 15 s, and 60°C for 1 min using the StepOnePlus Real-Time PCR System (Applied Biosystems). The cycle threshold (Ct) values, mRNA levels of reference, and target genes were determined by the relative quantification method, as specified by StepOne Software v 2.0 (Applied Biosystems). Standard curves were generated from five-fold serial dilutions of cDNA transcribed from gill mRNA samples. The expression levels of target genes were normalized to EF1α mRNA levels. Data are expressed as fold-change from FW values of 4-mo-old fish. The primers used are shown in Table 1.

**TABLE 1:** Primer sets used in real-time qPCR.

Gene name	GenBank accession no		Primer sequence 5′ to 3′	Efficiency
NKAα1a	LC556924	Forward	AAC​TGA​TTT​GGT​CCC​TGC​AA	114.2%
		Reverse	ATG​CAT​TTC​TGG​GCT​GTC​TC	
NKCC1a	AY513737	Forward	GGA​GGC​AAG​ATC​AAC​AGG​ATT​G	92.8%
		Reverse	AAT​GTC​CGA​AAA​GTC​TAT​CCT​GAA​CT	
NCC2	EU518934	Forward	CCG​AAA​GGC​ACC​CTA​ATG​G	113.5%
		Reverse	CTA​CAC​TTG​CAC​CAG​AAG​TGA​CAA	
EF1α	LC556928	Forward	AGC​AAG​TAC​TAC​GTG​ACC​ATC​ATT​G	84.6%
		Reverse	AGT​CAG​CCT​GGG​AGG​TAC​CA	

### Radioimmunoassays

Prl_188_ levels in Prl cell culture medium was measured by homologous RIA as previously described (Ayson et al., 1993; Yada et al., 1994; Yamaguchi et al., 2016), using a primary antibody (antiserum) raised in rabbit against Prl_188_ (anti-tPrl_188_), and a secondary antibody raised in goat against rabbit IgG (anti-rabbit IgG; Sigma).

### Statistical Analysis

Gene expression of effectors of ion transport from transfer experiments, reported as a fold-change from FW 4-mo-old groups, were analyzed by two-way ANOVA with age and salinity as main effects. Significant (*p* < 0.05) main and interaction effects were followed up with protected Fisher’s LSD. Prl_188_ release from cells incubated in hyposmotic media were expressed as fold-change relative to release from Prl cells incubated in hyperosmotic media, and analyzed by an unpaired t test at each age. The effects of age on branchial mRNA expression in response to oPrl were reported as a fold-change from controls (no added oPrl), and analyzed by two-way ANOVA with age and oPrl treatment as main effects. Significant (*p* < 0.05) main effects of treatment and age were followed up by a Dunnett’s test and a t test, respectively. Data are expressed as mean ± SEM. All statistical analyses were performed using GraphPad Prism 6 (GraphPad, San Diego, CA).

## Results

### Effect of Age on Survival Following Salinity Transfer

**FIGURE 1:**
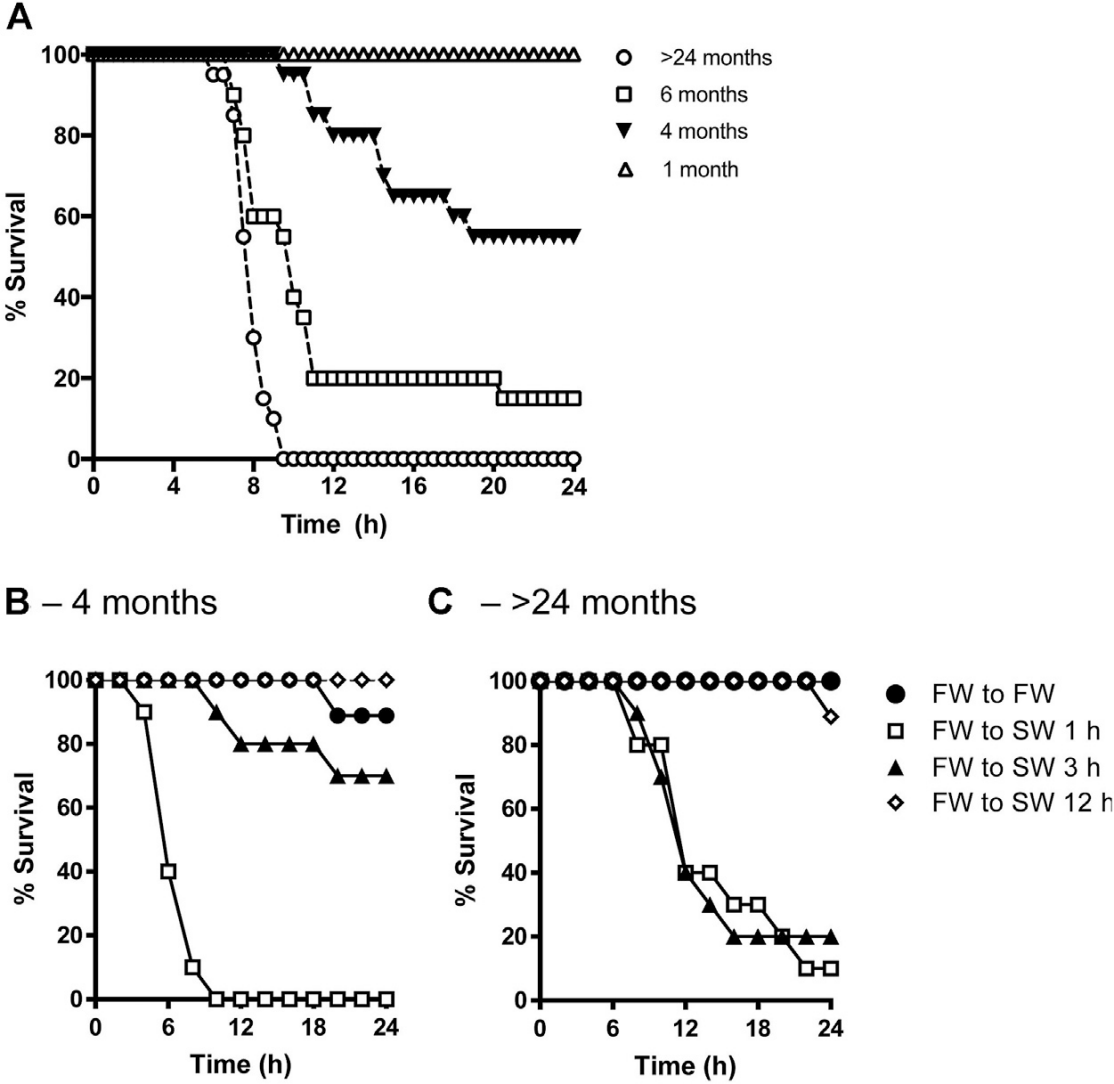
**(A)** Effects of age on percent survival of tilapia transferred from FW to SW (n = 20). Adult tilapia aged 4 months **(B)** or >24 months **(C)** were then kept in FW (filled circles) or transitioned from FW to SW over a 1-h (open squares), 3-h (filled triangles), and 12-h (open diamonds) period (*n* = 10).

There were no survivors aged >24 mo by 9.5 h following transfer from FW to SW. On the other hand, 15, 55, and 100% of 6-, 4-, and 1-mo-old fish, respectively, survived the salinity transfer by 24 h (Figure 1A). In a separate trial, when 4-mo-old tilapia were transferred from FW to SW over a 1-, 3-, and 12-h period, 0, 70, and 100% of the fish survived by 24 h, respectively (Figure 1B). By contrast, the survival rates of fish aged >24 mo transferred from FW to SW over a 1-, 3-, and 12-h period were 10, 20, and 90% by 24 h, respectively (Figure 1C).

### Effects of Age on Salinity-Dependent Branchial Gene Expression of Ion Transporters and Pumps

**FIGURE 2:**
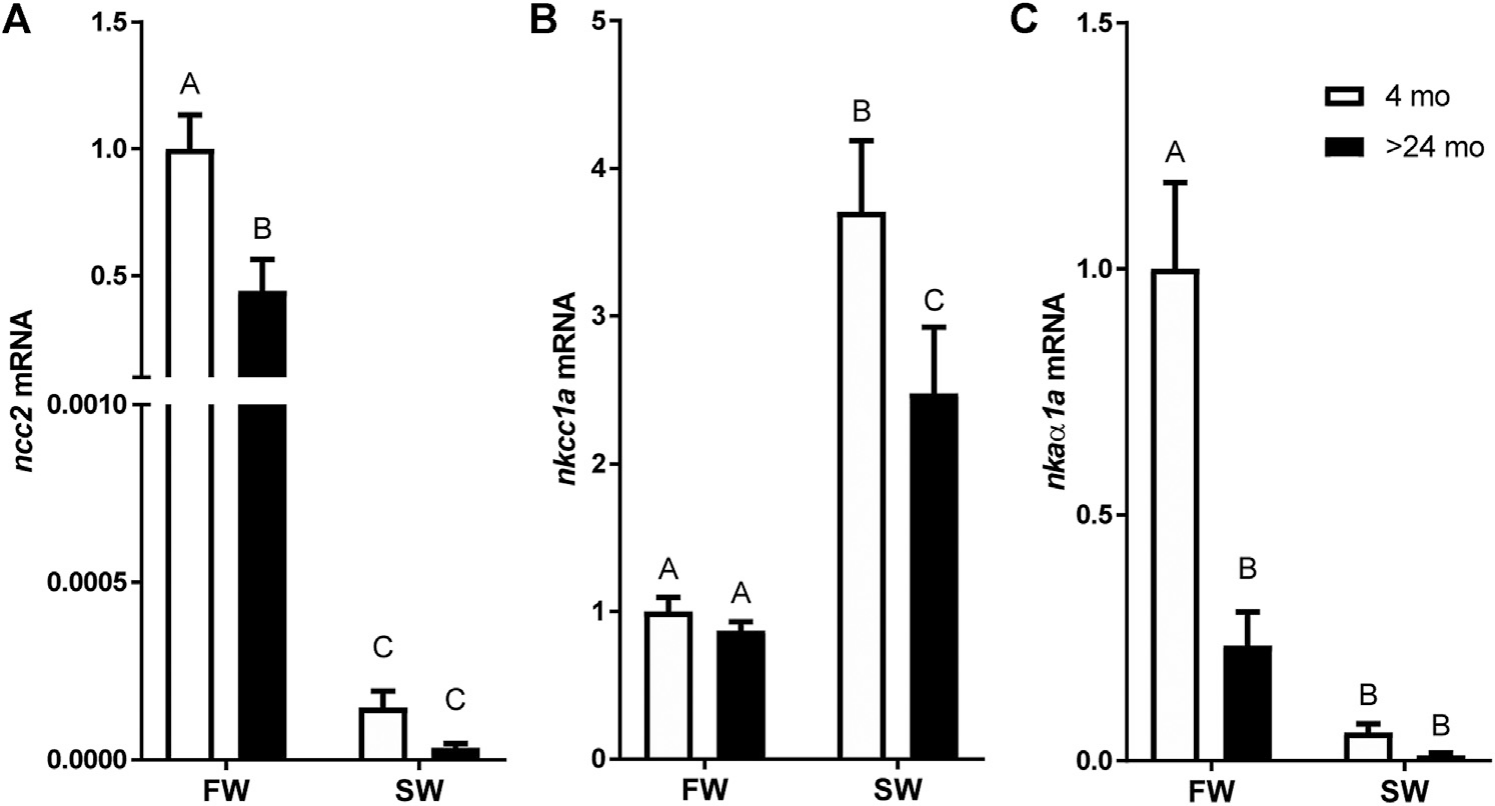
Effects of age and salinity on the expression of branchial mRNA expression of **(A)** *ncc2*, **(B)** *nkcc1a*, and **(C)** *nkaα1a.* White and black bars represent means from 4-mo to >24-mo fish. Fish were sampled at the end of the 24-h period following transfer to FW or a 12-h transition to SW. Means not sharing the same letter are significantly different at *p* < 0.05.

A two-way ANOVA revealed significant effects of salinity and age on the expression of all genes. Significant interactions were found in the expression of *ncc2* and *nkaα1a* but not in *nkcc1a*. The *ncc2* expression levels of tilapia aged 4 and >24 mo were markedly decreased by 24 h of transfer from FW to SW. While there was no significant difference between ages in fish acclimated to SW, *ncc2* expression was higher in fish aged 4 mo than those aged >24 mo (Figure 2A). By contrast, *nkcc1a* expression was upregulated following SW transfer. The rise in *nkcc1a* expression in response to SW transfer was greater in the younger fish, whereas no significant difference was detected between ages in FW-acclimated fish (Figure 2B). Similar to the patterns observed for *ncc2* expression, *nkaα1a* expression was considerably higher in FW-acclimated 4-mo-old fish when compared with SW-acclimated or >24-mo-old fish (Figure 2C).

### Effects of Age on Hyposmotically Induced Prolactin Release

**FIGURE 3:**
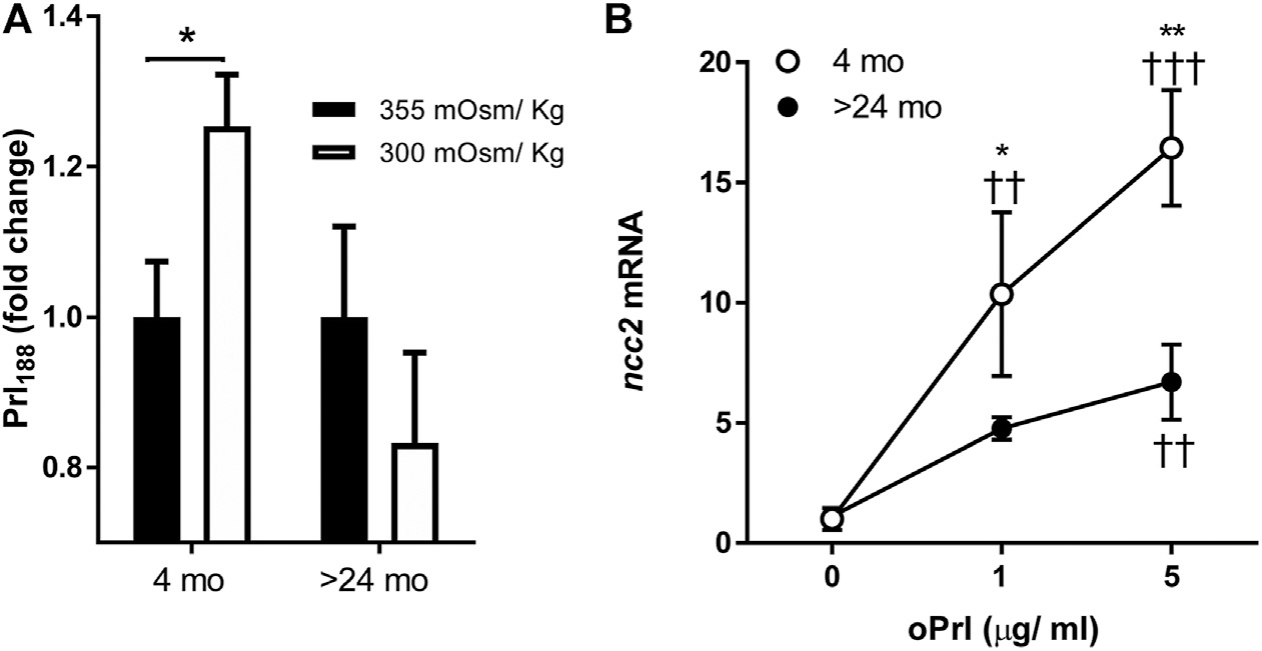
**(A)** Effects of age (4 months vs >24 months) on hyposmotically induced Prl_188_ release from dispersed Prl cells of FW-acclimated fish incubated for 1 h. Black and white bars represent hyperosmotic (355 mOsm/kg) and hyposmotic (300 mOsm/kg) media, respectively. * significantly different from each other at *p* < 0.05. **(B)** Effect of oPrl on the expression of *ncc2* in gill filaments of FW-acclimated tilapia incubated overnight. Open and filled circles represent means from 4- to >24-mo-old fish, respectively. *, ** significantly different between ages at *p* < 0.05 and *p* < 0.01, respectively; ††, ††† significantly different from 0 dose at *p* < 0.01 and *p* < 0.001, respectively.

Hyposmotically-induced Prl_188_ release was observed by 1 h of incubation of dispersed Prl cells from tilapia aged 4 mo but not from those aged >24 mo (Figure 3A).

### Effect of Ovine Prl on Branchial Expression of *ncc2*

We tested the age-dependent transcriptional responses of gill filaments to different concentrations of oPrl over an 18-h incubation period. The expression of *ncc2* significantly increased in response to oPrl in a concentration-dependent manner in tilapia of both ages (Figure 3B). The response of 4-mo-old tilapia to oPrl at both concentrations, however, was more robust than that of >24-mo-old tilapia. The greatest difference between ages was observed in gill filaments exposed to 5 μg/ml of oPrl; at that concentration, expression of *ncc2* was 16.4- and 6.0-fold higher relative to controls in 4- and >24-mo-old tilapia, respectively.

## Discussion

We characterized the effects of age on salinity tolerance in Mozambique tilapia. The survival rate of tilapia at various ages was examined after rapid (1 h) and delayed changes in environmental salinity. Overall, the survival rates after direct transfer from FW to SW were inversely related with age (Figure 1A). The results are generally consistent with previous salinity transfer trials, where adults typically require acclimation to an intermediate salinity or a changing salinity regime prior to full transfer to SW (Seale et al., 2002; Moorman et al., 2015). When tilapia were transferred from FW to SW over longer periods, the survival rates were higher in 4-mo-old fish compared with >24-mo-old fish by 3 h, indicating that older fish require a longer acclimation period during a salinity challenge (Figures 1B,C). Nevertheless, some discrepancies in survival rates of both 4-mo- and >24-mo-old fish during the direct transfer (1 h) from FW and SW were observed between trials. As a direct salinity challenge imparts considerable physiological stress (Wendelaar Bonga, 1977), there could be individual differences in fitness that may account for these discrepancies in survival rates. It is worth noting that Mozambique tilapia grows at slower rates with age, especially >24-mo and up to 14 years (Tachihara and Obara, 2003), and the metabolic rate increases in response to a rise in environmental salinity (Zikos et al., 2014). Moreover, unlike mammals, growth in fishes is generally indeterminate as it is largely dependent on environmental conditions (Dutta, 1994). Thus, within a given cohort, body sizes can vary widely, and in turn pose an additional challenge to osmoregulation, especially in smaller fish which have larger surface to volume ratios. In the current trials, despite their smaller size, younger fish had greater tolerance to a rise in salinity, suggesting that the observed decline in salinity tolerance in >24-mo-old fish is tied to age rather than size.

Irrespective of body size, plasma osmolality is elevated with increasing salinities, typically rising up to ∼440 mOsm/kg in surviving Mozambique tilapia transferred from FW to brackish water (28–30 ppt), and up to ∼550 mOsm/kg following a direct and lethal transfer to SW (Seale et al., 2012a; Moorman et al., 2015). While baseline plasma osmolalities may vary individually, there are no distinctive patterns across a wide range of body sizes (Moorman et al., 2015; Pavlosky et al., 2019). During a direct one-way transfer from FW to SW, the capacity of Mozambique tilapia to survive becomes largely compromised. Together, both trials indicate that older fish are less capable of recovering a salinity challenge, and require a transition period greater than 3 h to survive the transfer between FW and SW by 24 h. Because fish sizes typically increase with age, we also compared osmoregulatory processes between fish of different age-groups through *in vitro* approaches, where tissue quantity can be normalized across body sizes.

To examine whether the age-dependent loss in ability to acclimate to SW was associated with osmoregulatory capacity, gills were sampled from young and old fish that survived the 24-h challenge. In gills, ionocytes are responsible for ion uptake in FW and ion secretion in SW (Evans et al., 2005; Foskett et al., 1983). Active ion secretion in the gill of SW-acclimated fish is mediated by Nkcc1a in the basolateral membrane of ionocytes (Hwang et al., 2011), whereas ion uptake are largely mediated by Ncc2 in the apical membrane and Nkaα1a in the basolateral membrane when fish are in FW (Hiroi et al., 2008; Inokuchi et al., 2008; Tipsmark et al., 2011). Consistent with its function in ion uptake, branchial *ncc2* and *nkaα1a* expression was higher in FW-acclimated fish than those in SW (Figures 2A,C). By contrast, branchial expression of *nkcc1a* was higher in SW fish (Figure 2B). Notably, the relative expression of *nkcc1a*, which plays an important role in hyposmoregulation, was significantly upregulated in younger fish after SW transfer. This finding indicates a clear effect of age in the sensitivity of branchial ionocytes. Conversely, salinity-induced transcriptional activation of ion transport was attenuated in older fish facing a SW challenge.

Next, we investigated whether the age of fish affected the capacity of dispersed Prl cells from the RPD of tilapia pituitaries to respond differently to a hyposmotic stimulus. Tilapia Prl cells produce two Prl molecules: Prl_188_ and Prl_177_. Prl_188_ was measured in this experiment as it is more sensitive to hyposmotic stimuli than Prl_177_ (Seale et al., 2012a). Prl_188_ release has been consistently shown to increase in response to a fall in extracellular osmolality *in vitro* (Dharmamba and Nishioka, 1968; Ingleton et al., 1973; Grau et al., 1981; Richman et al., 1986; Seale et al., 2002; Seale et al., 2012a). In the current study, Prl cells from 4-mo-old tilapia increased Prl_188_ release in response to a physiologically relevant hyposmotic stimulus, 300 mOsm/kg, while cells from tilapia aged >24 mo did not (Figure 3A). The lack of responsiveness of older tilapia is analogous to the lower osmotic sensitivity of Prl cells of Nile tilapia (*O. niloticus*), a congener of lower salinity tolerance (Yamaguchi et al., 2018). As a potent hyperosmoregulatory hormone in fish, Prl acts by promoting ion uptake and reducing water permeability in gill (Hirano, 1986). Specifically, oPrl has been shown to upregulate branchial *ncc2* and *nka α1a* in Mozambique tilapia; *nkcc1a* was less responsive to oPrl (Breves et al., 2010; Inokuchi et al., 2015). We focused on *ncc2* because it encodes an apical membrane protein that functions to directly absorb ions from environmental water. The concentration-dependent, oPrl-induced upregulation of *ncc2* in gill filaments incubated for 18 h was more pronounced in 4-mo-old tilapia than in >24-mo-old fish (Figure 3B). This result supports the notion that branchial ionocytes loose sensitivity to both osmotic and endocrine stimuli with age.

In conclusion, for the first time, we experimentally addressed the age-related loss in the sensitivity of osmoregulatory transcripts associated with a transfer from FW to SW in an euryhaline teleost. Moreover, the capacity of Prl cells to respond to hyposmotic stimuli and of gill filaments to respond to oPrl *in vitro* was suppressed in older fish. Our findings indicate that multiple aspects of osmotic homeostasis, from osmoreception to hormonal and environmental control of osmoregulation, appear to decline in direct relation to age and adversely affect the ability of older fish to survive a transfer to hyperosmotic environments. Future studies aimed at comparing different size classes within the same age cohort could further resolve whether there are also size-specific effects on osmoregulatory capacity.

## Data Availability

The datasets presented in this study can be found in online repositories. The names of the repository/repositories and accession number(s) can be found in the article/Supplementary Material.
